# Maternal dietary pattern and its association with birthweight in Northern Ethiopia: A hospital‐based cross‐sectional study

**DOI:** 10.1002/fsn3.3368

**Published:** 2023-04-11

**Authors:** Selamawit Gebreyohannes Weldegebriel, Selemawit Asfaw Beyene, Freweini Gebrearegay Tela, Zenawi Hagos Gufue, Helen Teweldebrhan Hailu

**Affiliations:** ^1^ Department of Public Health, College of Medicine and Health Sciences Adigrat University Tigray Ethiopia; ^2^ Department of Nutrition and Dietetics, School of Public Health, College of Health Sciences Mekelle University Tigray Ethiopia

**Keywords:** birthweight, dietary pattern, Northern Ethiopia, pregnant mother

## Abstract

Birthweight is a useful public health measure of maternal health, nutrition, healthcare delivery, and child morbidity and mortality. Previous research did not focus on dietary patterns but rather on a single or a few foods or nutrients. This study aimed to assess the maternal dietary pattern and its association with birthweight in northern Ethiopia. A hospital‐based cross‐sectional study was conducted among 373 pregnant mothers in their third trimester of pregnancy who came to attend their routine antenatal care service. The food frequency questionnaire was collected from the previous week, and the birthweight data were collected from the medical records after delivery. Three maternal dietary patterns were identified; dietary pattern includes eggs, milk, milk products, and certain fruits and roots. Dietary pattern 2 includes certain vegetables, green leafy vegetables, vitamin A‐rich vegetables, pulses such as beans, peas, and chickpeas, and drinks like coffee, tea, and soda. Dietary pattern 3 includes meat, nuts, and grains such as teff, corn, wheat, and white flour. Dietary pattern 1 (*β* = 52.45, *p* = .03) and dietary pattern 2 (*β* = 66.76, *p* = .01), residency (*β* = 287.08, *p* < .001), a mid‐upper‐arm circumference of 21–23 cm (*β* = 187.10, *p* = .02), a mid‐upper‐arm circumference of >23 cm (*β* = 272, *p* = .01), and gestational age at delivery (*β* = 12.58, *p* = .004) were the factors significantly associated with increased birthweight. The maternal dietary pattern has a significant association with birthweight. The focus should be given to maternal dietary patterns to prevent suboptimal and high birthweight.

## Graphical abstract

Complex interactions between nutrients are not taken into account by the single‐nutrient analysis, and it is challenging to analyze nutrient impacts separately. According to our research, the food patterns of pregnant women in their third trimester of pregnancy can be best explained by three unique dietary patterns. A higher birthweight result was observed in women who ingested more eggs, milk and milk products, pulses, vegetables and roots, fruits, beverages, and sweets. For the majority of the time, food habits usually remain steady during pregnancy, and there is a strong correlation between the maternal dietary pattern and birthweight.

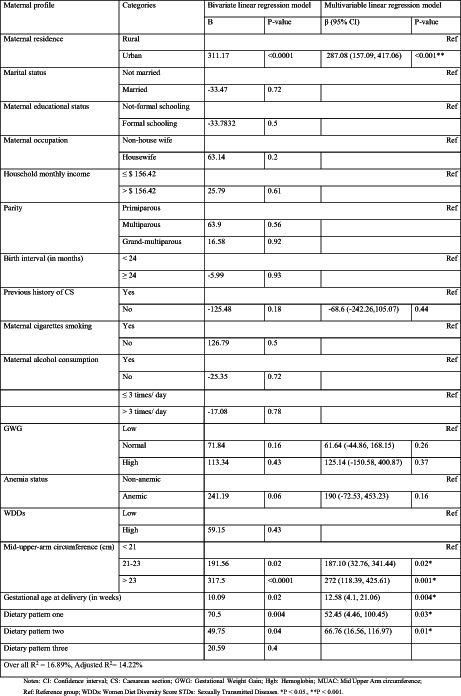

## 1 INTRODUCTION

Energy and nutrient needs are typically higher during pregnancy to support the mother's increased metabolism, blood volume, and red cell mass expansion, which helps the mother to deliver nutrients to the fetus (Kliegman et al., 2016). During the third trimester of pregnancy, in particular, energy, and micronutrients increase exceptionally (Barker, 1995). Given that it depends on maternal health and nutrition throughout pregnancy, access to healthcare, and poverty, an infant's birthweight is a useful public health measure of maternal health (McGuire & World Health Organization, 2015).

Around the world, over a million infants die in the first month of life, with Sub‐Saharan Africa (SSA) having a mortality rate of 50% within 1 day after delivery. Specifically, in Ethiopia, 1 in every 35 neonates dies within the first month (Central Statistical Agency, Ethiopia, and ICF. Ethiopia Demographic and Health Survey 2016, 2017). Worldwide, low birthweight contributes to 60–80% of all neonatal deaths (McGuire & World Health Organization, 2015). Low birthweight is associated with increased childhood and adulthood malnutrition, and certain adulthood chronic disorders such as hypertension, diabetes, and coronary heart disease (Barker, 1995).

Nutritionists are increasingly using dietary pattern assessment because it recognizes the overall dietary pattern that accounts for the interaction (synergistic or antagonistic) effects of nutrients (Smolin & Grosvenor, 2013). Improving maternal nutritional status by advancing maternal dietary patterns instead of a single nutrient approach has better benevolence (Chia et al., 2019). Thus, there is conflicting evidence regarding the health benefits of particular nutrients for women who are fertile (Smolin & Grosvenor, 2013). Studies that focus on specific nutrients or foods offer advice on how to diversify your diet, and analyze your eating patterns frequently fall short of this goal (Slattery, 2010).

A single or a small number of foods or nutrients were the major focus of earlier investigations to determine the connection between maternal nutrition and birthweight (Slattery, 2010). The single‐nutrient method, however, has several drawbacks (Northstone et al., 2008). Instead of eating isolated nutrients, people consume meals made up of a variety of foods; single‐nutrient analysis does not take into account complex interactions between nutrients, and nutrient impacts are challenging to examine separately by single‐nutrient analysis (Slattery, 2010).

Using dietary patterns rather than isolated nutrients would be better for understanding the gap in maternal and child morbidity, and mortality related to malnutrition (Chia et al., 2019). As the previous studies were from Western countries, there is limited data available regarding the association between maternal dietary patterns and neonatal birthweight in Africa, including in Ethiopia (Abubakari & Jahn, 2016). Evaluation and a better understanding of maternal dietary patterns will improve the birthweight outcomes, maternal and child morbidity, mortality, and complications, and improves the quality of care given in nutritional counseling for pregnant mothers. This study was intended to assess the maternal dietary pattern and its association with birthweight in Northern Ethiopia.

### 1.1 Key messages

- Complex interactions between nutrients are not taken into account by the single‐nutrient analysis, and it is challenging to analyze nutrient impacts separately.
- According to our research, the food patterns of pregnant women in their third trimester of pregnancy can be best explained by three unique dietary patterns.
- A higher birthweight result was observed in women who ingested more eggs, milk and milk products, pulses, vegetables and roots, fruits, beverages, and sweets.
- For the majority of the time, food habits usually remain steady during pregnancy, and there is a strong correlation between maternal dietary pattern and birthweight.

## 2 METHODS

### 2.1 Study area and period

This study was conducted in Adigrat general hospital, a government‐owned general hospital and teaching center for Adigrat University. It also serves as a referral hospital for surrounding catchment areas in the Eastern Zone of the Tigray and the Afar region with 142,765 patient flows per year. Based on the 2019 annual report, there were a total of 4244 pregnant mothers on their first antenatal care (ANC) visit, 1188 on their fourth ANC visit, and a total of 2852 deliveries. The study was conducted from March 1 to May 29, 2020.

### 2.2 Study design and participants

A hospital‐based cross‐sectional study was conducted among pregnant women who were in their third trimester of pregnancy (>37 weeks of gestation) coming to attend their routine antenatal care service and their respective live‐born baby medical records in Adigrat general hospital, Tigray, Ethiopia. Mothers with ultrasound‐confirmed multiple pregnancies, who were mentally unstable and critically ill, mothers with pregnancy‐induced hypertension, and gestational diabetes mellitus were excluded from the study since they need special dietary intake.

### 2.3 Sample size and sampling technique

The sample size was calculated using a single population proportion formula with the assumption of 95% CI and a 5% margin of error. The proportion of pregnant mothers who ate an unhealthy dietary pattern, *p* = 28.3%, was taken (Abubakari & Jahn, 2016). With these presumptions, the total sample size become 312, and when a 15% non‐response rate was added, the ultimate sample size increased to 373. A consecutive sampling technique was used to select eligible mothers coming for their routine antenatal care visit to the hospital.

### 2.4 Data collection tool and procedure

A pre‐tested interviewer‐administered semi‐structured questionnaire was used to collect data and dietary history was taken using the Women Diet Diversity Scores (WDDs) and Food Frequency Questioner (FFQ). WDDs were collected using 24‐h dietary recall intake history taken from the Food and Agricultural Organization (FAO) guideline. The FAO guideline was translated to the local language, Tigrigna, and then translated back to English to maintain its consistency, and no frequent food types were removed.

The dietary history was taken from the previous day of the data collection day. The dietary pattern was assessed using the modified Hellen FFQ containing 43 food types (Persson et al., 1998). Frequencies of consumption were taken by a recall of dietary intakes of the previous week from the data collection day. Anthropometric measurements of the mothers were assessed by using the mid‐upper‐arm circumference (MUAC) and weight.

A digital weight scale was used to measure maternal weight. First, the scale was zeroed before stepping on them, the mother was asked to step onto the center of the scale and to stand still, then we waited until the weight was displayed and remained fixed in the display panel, and read aloud the weight to the nearest 0.1 kg. Weight before pregnancy was self‐reported but if she does not remember, it was taken retrospectively from her medical record. MUAC was measured using a measuring tape and recorded accurately to the nearest 0.1 mm.

Laboratory measurement for hemoglobin level was also recorded from the laboratory result papers. Secondary data on the birthweight of their respective live births were taken from their medical record after delivery. Six data collectors who work at the health institution (midwifery, nurses, and health officers) and one supervisor collected the data. The birthweight of the newborn was the main outcome variable, the independent variables were the sociodemographic characteristics, reproductive characteristics, medical and risky behavior characters, and nutritional factors.

### 2.5 Data management and analysis

The collected data were coded and checked for consistency and completeness up to the end of each data collection period. The data were entered using Epidata Manager version 4.6.0.2 and exported to the Statistical Package for Social Sciences (SPSS) software version 25 for Windows. The descriptive statistics of numeric variables were presented using median and interquartile range (IQR), and frequency and percentages were used for categorical variables.

Principal component analysis (PCA) was used to generate dietary patterns with varimax rotation. The 43 food types that were collected using FFQ of 1‐week duration were grouped into 10 food groups before doing PCA. To maximize each variable's loading on one of the extracted components while minimizing its loading on all other factors, the variance was based on rotated sums of squared loading, and the varimax with Keiser normalization was employed as the orthogonal rotation approach.

Strong associations were defined as factor loadings with a magnitude >0.3 (Denova‐Gutiérrez et al., 2011; Esmaillzadeh et al., 2007; Kim & Mueller, 1978; Newby et al., 2006), and foods within a factor with loadings of these levels were regarded as descriptive of the dietary pattern associated with that factor. The association between dietary patterns and birthweight was assessed using a linear regression model. The assumptions of linearity, independence, normality, multi‐collinearity, and homoscedasticity were checked before running the model. The variables with *p*‐value <.2 in the bivariate linear regression model were entered into the multiple linear regression model. An adjusted *β* with its 95% CI was presented, and a *p* < .05 and was used to declare the statistical significance. An adjusted *R* ^2^ was taken to determine the proportion of variation that was explained by the regression model.

### 2.6 Operational definitions

Anemic mothers were mothers with a hemoglobin level of less than 11 g/dL (World Health Organization, 2001). The WDDs were grouped as low (<5) and high (≥5) (Schwei et al., 2017). Total weight gain during the entire pregnancy was categorized as <11 kg (low), 11–16 kg (normal), and >16 kg (high). Maternal gravidity classification was as primigravida (no pregnancies), multigravida (2–4 pregnancies), and grand‐multigravida (≥5 pregnancies). Maternal parity classification was as primiparous (no birth), multiparous (2–4 births), and grand multiparous (≥5 births) (Tela et al., 2019).

### 2.7 Ethics approval

All pregnant mothers provided written informed consent. Ethical approval was obtained from the Health Research Ethics Review Committee (HRERC) of THE College of Health Sciences, Mekelle University (ERC 1553/2020). The data collected from the medical records were handled with strict confidentiality; neither the case records nor the data extracted were used for any other purpose, and all information collected from pregnant mothers was stored anonymously. This research was carried out following the Helsinki Declaration of 1964.

## 3 RESULTS

### 3.1 Sociodemographic characteristics

Of the 373 study participants, 354 pregnant mothers completed the study yielding a response rate of 94.91%. Nineteen (5.09%) mothers were excluded from the study because sixteen mothers were lost before delivery and three mothers had a stillbirth. The median age of the study participants was 26 years with an IQR (23–31) years. Two hundred ninety‐nine (84.46%) pregnant mothers were urban residents and 327 (92.37%) pregnant mothers were married (Table 1).

**TABLE 1:** Sociodemographic characteristics of pregnant women receiving routine antenatal care service in Northern Ethiopia, 2020 (*n* = 354).

Maternal profile	Categories	Frequency (%)
Age, median (interquartile range), years		26 (23–31)
Household monthly income, median (interquartile range), US$		149 (89.38–208.56)
Age group (in years)	≤24	125 (35.31)
	25–29	108 (30.51)
	30–34	63 (17.8)
	35–39	47 (13.28)
	≥ 40	11 (3.1)
Residence	Urban	299 (84.46)
	Rural	55 (15.54)
Marital status	Married	327 (92.37)
	Not married, living together	18 (5.08)
	Others^a^	9 (2.55)
Religion	Orthodox	320 (90.4)
	Catholic	21 (5.93)
	Muslim	13 (3.67)
Maternal educational status	Unable to read and write	20 (5.65)
	Able to read and write	112 (31.64)
	Primary school completed	138 (38.98)
	Secondary school completed	13 (3.67)
	Diploma and above	71 (20.06)
Maternal occupational status	Housewife	188 (53.11)
	Farmer	15 (4.24)
	Merchant	58 (16.38)
	Government employees	74 (20.9)
	Non‐governmental employees	8 (2.26)
	Others^b^	11 (3.11)
Husband's educational level (*n* = 352)	Unable to read and write	15 (4.26)
	Able to read and write	99 (28.13)
	Primary school completed	136 (38.64)
	Secondary school completed	13 (3.69)
	Diploma and above	89 (25.28)
Husband's occupational status (*n* = 352)	Farmer	29 (8.24)
	Merchant	88 (25)
	Government employee	101 (28.69)
	Non‐governmental employee	16 (4.55)
	Self‐employed	109 (30.97)
	Unemployed	9 (2.55)
Household monthly income (In US$^c^)	≤156.42	224 (63.28)
	156.45–232.4	54 (15.25)
	232.43–324.76	60 (16.95)
	≥324.79	16 (4.52)

^a^ Single and divorced.

^b^ Student and self‐employed.

^c^ According to the National Bank of Ethiopia, on average, 1 US$ was exchanged for 33.5631 Ethiopian birrs during the data collection period from March 1 to May 29, 2020.

### 3.2 Reproductive health characteristics, medical, and risky behavioral factors

The mean birthweight of the newborn was 3125 g with a standard deviation of (±460) g. Three hundred thirteen (88.42%) mothers were multigravida while 198 (84.26%) of the mothers were multiparous, with a median birth interval of 24 months IQR 17.5–36 months. Almost all (98.31%) of the pregnant mothers were not ever cigarette smokers. Concerning the pregnant mother's serostatus, 27 (7.63%) of them were human immunodeficiency virus positive. Thirteen (3.67%) and four (1.13%) mothers were diagnosed with sexually transmitted diseases (STDs) and malaria during their current pregnancy, respectively. Concerning the mode of delivery of the pregnant mothers, 54 (15.25%) gave birth by cesarean section, while the rest gave birth by spontaneous vaginal delivery (Table 2).

**TABLE 2:** Reproductive health characteristics, medical, and risky behavioral factors of pregnant women receiving routine antenatal care service in Northern Ethiopia, 2020 (*n* = 354).

Maternal profile	Categories	Frequency (%)
Birthweight of the newborn, mean (standard deviation), in g		3125 (±460)
Age at first pregnancy, median (interquartile range), years		22 (20–24)
Gestational age at delivery, median (interquartile range), weeks		37.8 (36.3–39.4)
Birth interval, median (interquartile range), months		24 (17.5–36)
Age at first pregnancy (in years)	≤17	14 (3.95)
	18–28	321 (90.68)
	29–39	19 (5.37)
Gravidity	Primigravida	1 (0.28)
	Multigravida	313 (88.42)
	Grand‐multigravida	40 (11.30)
Parity (*n* = 235)	Primiparous	22 (9.36)
	Multiparous	198 (84.26)
	Grand‐multiparous	15 (6.38)
Birth interval (In months, *n* = 196)	<24	104 (53.06)
	≥24	92 (46.94)
Newborn birthweight categories	Low birthweight	29 (8.19)
	Normal birthweight	307 (86.72)
	Macrosomia	18 (5.09)
Gender of the newborn	Male	179 (50.56)
	Female	175 (49.44)
Mode of delivery of the newborn	Spontaneous vaginal delivery	300 (84.75)
	Cesarean delivery	54 (15.25)
Previous history of abortion	Yes	50 (14.12)
	No	304 (85.88)
Type of abortion faced (*n* = 47)	Spontaneous abortion	44 (93.62)
	Induced abortion	2 (4.26)
	Both	1 (2.12)
Previous history of cesarean section delivery (*n* = 322)	Yes	27 (8.39)
	No	295 (91.61)
Any intake of unprescribed medication during the current pregnancy	Yes	7 (1.98)
	No	347 (98.02)
Any symptoms of STDs during the current pregnancy	Yes	13 (3.67)
	No	341 (96.33)
Treatment of STDs that occurred during the current pregnancy (*n* = 14)	Treated	11 (84.62)
	Not treated	2 (15.38)
Malaria status during the current pregnancy	Yes	4 (1.13)
	No	350 (98.87)
HIV/AIDS serostatus	Positive	27 (7.63)
	Negative	327 (92.37)
Maternal history of smoking cigarettes	Yes	6 (1.69)
	No	348 (98.31)
History of passive smoking during the current pregnancy	Yes	13 (3.67)
	No	341 (96.33)
Maternal history of alcohol consumption	Yes	207 (58.47)
	No	147 (41.53)
History of alcohol consumption during the current pregnancy (*n* = 209)	Yes	147 (70.33)
	No	62 (29.67)
Frequency of alcohol consumption during the current pregnancy (*n* = 146)	Almost every day	3 (2.05)
	At least once a week	24 (16.44)
	Less than once a month	119 (81.51)

Abbreviations: HIV/AIDS, human immunodeficiency virus/acquired immune‐deficiency syndrome; STDs, sexually transmitted diseases.

### 3.3 Maternal nutritional status

During this study period, 65 (18.36%) of the pregnant mothers were eating fast foods. One hundred eighty‐eight (55.46%) of the pregnant mothers reported having received nutritional counseling from a health professional during the current pregnancy. Regarding the feeding frequency, 285 (80.51%) of the mothers ate greater than three times a day, and concerning the anemia status of the mothers, 341 (96.33%) had a hemoglobin level of ≥11 g/dL. In addition, 234 (88.64%) of the mothers were provided with iron–folic acid supplementations. The median gestational weight gain during the current pregnancy was 10.5 kg with IQR (Abubakari & Jahn, 2016; Denova‐Gutiérrez et al., 2011; Esmaillzadeh et al., 2007; Northstone et al., 2008; Persson et al., 1998) kg (Table 3).

**TABLE 3:** Nutritional status of pregnant women receiving routine antenatal care service in Northern Ethiopia, 2020 (*n* = 354).

Nutritional characteristics	Categories	Frequency (%)
Maternal hemoglobin level, median (interquartile range), g/dL		13.03 (12.3–13.7)
GWG, median (interquartile range), kg		10.5 (8–12)
WDDs, median (interquartile range), scores		4 (3–5)
Ever heard about nutrition during pregnancy	Yes	340 (96.05)
	No	14 (3.95)
Sources of information for the nutritional counseling during the current pregnancy	Television	110 (32.45)
	Radio	28 (8.26)
	Newsletters	13 (3.83)
	Health workers	188 (55.46)
Feeding frequency during pregnancy	≤3 times/ day	69 (19.5)
	>3 times/ day	285 (80.5)
Pregnant mothers who omitted food items during pregnancy	Yes	62 (17.51)
	No	292 (82.49)
Food taboo in the community for pregnant women	Yes	9 (2.54)
	No	345 (97.46)
Fasting during pregnancy	Yes	65 (18.36)
	No	289 (81.64)
Maternal nutritional supplementation during pregnancy	Supplemented	263 (74.29)
	Not supplemented	91 (25.71)
Nutritional supplementation type	Iron–folic acid	233 (88.6)
	Multivitamin	30 (11.36)
Maternal anemia status based on their Hgb level	Anemic	13 (3.67)
	Non‐anemic	341 (96.33)
GWG (in kg)	Low	218 (61.58)
	Normal	125 (35.31)
	High	11 (3.11)
WDDs	Low diet diversity score	312 (88.14)
	High diet diversity score	42 (11.86)
MUAC (in cm)	<21	43 (12.15)
	21–23	133 (37.57)
	>23	178 (50.28)

Abbreviations: GWG, gestational weight gain; Hgb, hemoglobin; MUAC, mid‐upper‐arm circumference; WDDs, Women Diet Diversity Scores.

### 3.4 Dietary patterns

After removing foods that were consumed less than 25% of the time, the food items were grouped into 10 food groups. According to our research, the food patterns of pregnant women in their third trimester of pregnancy can be best explained by three unique dietary patterns. They are labeled as pattern 1, pattern 2, and pattern 3, and these patterns explained 44.9% of the variability in the dietary pattern of pregnant women. Food groups with high factor loadings (0.3) on dietary pattern 1 included eggs, milk, and milk products, certain fruits (banana, orange, avocado, papaya, guava, and mango), and roots (potato and carrot).

Dietary pattern 2 was characterized by high factor loadings for certain vegetables (green leafy vegetables and vitamin A‐rich vegetables), pulses (beans, peas, and chickpeas), and drinks like coffee, tea, and soda. Dietary pattern 3 had higher loading factors for nuts, grains (teff, corn, wheat, and white flour), and meat. Dietary pattern 3 also had very low (negative) factor loadings for eggs, milk, and milk products, and pulses (Table 4).

**TABLE 4:** Loading values of the food groups consumed by pregnant women receiving routine antenatal care service in Northern Ethiopia, 2020 (*n* = 354).

Loading values			
	Patterns		
	One	Two	Three
Grains	−0.10	0.01	0.53
Nuts	0.02	−0.03	0.61
Roots	0.53	0.12	0.02
Vegetables	0.20	0.64	0.29
Pulses	0.07	0.77	−0.11
Fruits	0.51	−0.14	0.44
Egg	0.81	−0.16	−0.08
Milk and milk products	0.75	0.09	−0.06
Meat	0.03	0.17	0.45
Beverages	−0.16	0.63	0.05

### 3.5 Mean birthweight difference

There is a statistically significant difference in the newborn birthweight between urban and rural residents with *F*(1, 352) = 22.61, *p* ≤ .001. Similarly, there is a statistically significant difference in the newborn birthweight among pregnant mothers with different household monthly income categories, *F*(3, 350) = 2.77, *p* = .04. Moreover, there is a statistically significant difference in the newborn birthweight among pregnant mothers with different mid‐upper‐arm circumference measurements, *F*(2, 351) = 9.52, *p* = .001 (Table 5).

**TABLE 5:** One‐way analysis of variance for birthweight by different maternal profiles among pregnant women receiving routine antenatal care service in Northern Ethiopia, 2020 (*n* = 354).

Maternal profile		Sum of squares	Degree of freedom	Mean score	*F*‐statistic	*p*‐value
Age group	Between groups	88,590.6807	4	22,147.6702	0.10	.98
	Within groups	74,439,489.5	349	213,293.666		
	Total	74,528,080.2	353	211,127.706		
Maternal residence	Between groups	4,498,043.54	1	4,498,043.54	22.61	<.001**
	Within groups	70,030,036.7	352	198,948.968		
	Total	74,528,080.2	353	211,127.706		
Marital status	Between groups	430,155.497	3	143,385.166	0.68	.57
	Within groups	74,097,924.7	350	211,708.356		
	Total	74,528,080.2	353	211,127.706		
Maternal educational status	Between groups	544,503.288	4	136,125.822	0.64	.63
	Within groups	73,983,576.9	349	211,987.326		
	Total	74,528,080.2	353	211,127.706		
Maternal occupation	Between groups	1,488,923.07	6	248,153.846	1.18	.32
	Within groups	73,039,157.2	347	210,487.485		
	Total	74,528,080.2	353	211,127.706		
Household monthly income	Between groups	1,726,182.43	3	575,394.144	2.77	.04*
	Within groups	72,801,897.8	350	208,005.422		
	Total	74,528,080.2	353	211,127.706		
Gravidity	Between groups	27,473.1272	2	13,736.5636	0.06	.94
	Within groups	74,500,607.1	351	212,252.442		
	Total	74,528,080.2	353	211,127.706		
Parity	Between groups	104,367.828	2	52,183.914	0.22	.8
	Within groups	54,308,231.7	232	234,087.205		
	Total	54,412,599.5	234	232,532.476		
Birth interval	Between groups	1751.11462	1	1751.11462	0.01	.93
	Within groups	44,127,465.1	194	227,461.16		
	Total	44,129,216.2	195	226,303.673		
Previous history of CS	Between groups	389,494.013	1	389,494.013	1.82	.18
	Within groups	68,504,260.4	320	214,075.814		
	Total	68,893,754.4	321	214,622.288		
STDs during current pregnancy	Between groups	507,253.578	1	507,253.578	2.41	.12
	Within groups	74,020,826.7	352	210,286.439		
	Total	74,528,080.2	353	211,127.706		
Maternal history of smoking cigarettes	Between groups	94,820.6503	1	94,820.6503	0.45	.5
	Within groups	74,433,259.6	352	211,458.124		
	Total	74,528,080.2	353	211,127.706		
Maternal history of alcohol consumption during the current pregnancy	Between groups	28,023.0355	1	28,023.0355	0.13	.71
	Within groups	43,307,608.2	207	209,215.499		
	Total	43,335,631.2	208	208,344.381		
Feeding frequency during pregnancy	Between groups	16,212.0944	1	16,212.0944	0.08	.78
	Within groups	74,511,868.1	352	211,681.444		
	Total	74,528,080.2	353	211,127.706		
Maternal nutritional supplementation during pregnancy	Between groups	26.5998392	1	26.5998392	0.00	.99
	Within groups	74,528,053.6	352	211,727.425		
	Total	74,528,080.2	353	211,127.706		
Fasting during pregnancy	Between groups	6275.67059	1	6275.67059	0.03	.86
	Within groups	74,521,804.6	352	211,709.672		
	Total	74,528,080.2	353	211,127.706		
GWG	Between groups	491,010.385	2	245,505.193	1.16	.31
	Within groups	74,037,069.8	351	210,931.823		
	Total	74,528,080.2	353	211,127.706		
Anemia status	Between groups	728,481.126	1	728,481.126	3.47	.06
	Within groups	73,799,599.1	352	209,657.952		
	Total	74,528,080.2	353	211,127.706		
WDDs	Between groups	129,527.682	1	129,527.682	0.61	.43
	Within groups	74,398,552.5	352	211,359.524		
	Total	74,528,080.2	353	211,127.706		
MUAC	Between groups	3,833,270.16	2	1,916,635.08	9.52	.001*
	Within groups	70,694,810.1	351	201,409.715		
	Total	74,528,080.2	353	211,127.706		

Abbreviations: CS, cesarean section; GWG, gestational weight gain; MUAC, mid‐upper‐arm circumference; STDs, sexually transmitted diseases; WDDs, Women Diet Diversity Scores. *p < .05 and **p < .001.

According to the Tukey post hoc test analysis, there is a statistically significant difference in the newborn birthweight between pregnant mothers with a household monthly income of ≥$ 324.79 and those with $ 156.45–232.4, and mothers with a household monthly income of $ 156.45–232.4 had low‐birthweight babies than those with ≥$ 324.79 (*p* = .04), mean difference of − 358.8 (95% CI −703.24, −14.35). Similarly, there is a statistically significant difference in the newborn birthweight between pregnant mothers with MUAC <21 and 21–23 cm; mothers with MUAC <21 cm had low‐birthweight babies than those with MUAC 21–23 cm (*p* = .04), mean difference of − 191.56 (95% CI −380.94, −2.17).

Pregnant mothers with MUAC <21 cm had low‐birthweight babies than those with MUAC >23 cm; this was found to be statistically significant with *p* < .001 and mean difference of −317.5 (95% CI −500.95, −134.06). In addition, there is a statistically significant difference in the newborn birthweight between pregnant mothers with MUAC >23 cm and those with 21–23 cm, and mothers with MUAC 21–23 cm had low‐birthweight babies than those with MUAC >23 cm (*p* = .04), mean difference of 125.95 (95% CI 2.21, 249.68) (Table 6).

**TABLE 6:** Multiple comparisons for birthweight and maternal characteristics among pregnant women receiving routine antenatal care service in Northern Ethiopia, 2020 (*n* = 354).

Outcome variable: Newborn birthweight						
Tukey HSD		Mean difference	Std. error	Sig.	95% Confidence interval	
					Lower bound	Upper bound
**Household monthly income**	**Household monthly income**					
≤$ 156.42	≥24.79	227.67	118.02	0.33	−85.48	540.82
	156.45–232.4	−131.12	69.14	0.35	−314.58	52.33
	232.43–324.76	1.43	66.3	1.00	−174.48	177.34
≥$ 324.79	≤$ 156.42	−227.67	118.02	0.33	−540.82	85.48
	$ 156.45–232.4	−358.8	129.82	0.04*	−703.24	−14.35
	$ 232.43–324.76	−226.24	128.32	0.47	−566.73	114.24
$ 156.45–232.4	≤$ 156.42	131.12	69.14	0.35	−52.33	314.58
	≥$ 324.79	358.8	129.82	0.04	14.35	703.24
	$ 232.43–324.76	132.55	85.55	0.73	−94.44	359.54
$ 232.43–324.76	≤$ 156.42	−1.43	66.3	1.00	−177.34	174.48
	≥$ 324.79	226.24	128.32	0.47	−114.24	566.73
	$ 156.45–232.4	−132.55	85.55	0.73	−359.54	94.44
MUAC	MUAC					
<21 cm	21–23 cm	−191.56	78.73	0.04*	−380.94	−2.17
	>23 cm	−317.5	76.26	<0.001**	−500.95	−134.06
21–23 cm	<21 cm	191.56	78.73	0.04*	2.17	380.94
	>23 cm	−125.95	51.44	0.04*	−249.68	−2.21
> 23 cm	<21 cm	317.5	76.26	<0.001**	134.06	500.95
	21–23 cm	125.95	51.44	0.04*	2.21	249.68

*Note*: The mean difference is significant at the *p < .05 and **p < .001.

### 3.6 Independent effect of dietary patterns on birthweight

Among the three extracted dietary patterns of the pregnant mothers during their third trimester of pregnancy, dietary pattern 1 and dietary pattern 2 were significantly associated with higher birthweight while dietary pattern 3 was not significantly associated. After controlling the effect of other covariates, a 1 unit increase in the loadings of dietary pattern 1 was associated with a 52.45 g increase in birthweight, *β* = 52.45 (95% CI 4.46, 100.45). Similarly, a 1 unit increase in the loadings of dietary pattern 2 was associated with a 66.76 g increase in birthweight, *β* = 66.76 (95% CI 16.56, 116.97) (Table 7).

**TABLE 7:** The association between maternal factors and birthweight among pregnant women receiving routine antenatal care service in Northern Ethiopia, 2020 (*n* = 354).

Maternal profile	Categories	Bivariate linear regression model		Multivariable linear regression model	
		Β	*p*‐value	*β* (95% CI)	*p*‐value
Maternal residence	Rural	Ref			
	Urban	311.17	<.0001	287.08 (157.09, 417.06)	<.001**
Marital status	Not married	Ref			
	Married	−33.47	.72		
Maternal educational status	Not formal schooling	Ref			
	Formal schooling	−33.7832	.5		
Maternal occupation	Non‐house wife	Ref			
	Housewife	63.14	.2		
Household monthly income	≤$ 156.42	Ref			
	>$ 156.42	25.79	.61		
Parity	Primiparous	Ref			
	Multiparous	63.9	.56		
	Grand‐multiparous	16.58	.92		
Birth interval (in months)	<24	Ref			
	≥24	−5.99	.93		
Previous history of CS	Yes	Ref			
	No	−125.48	.18	−68.6 (−242.26,105.07)	.44
Maternal cigarettes smoking	Yes	Ref			
	No	126.79	.5		
Maternal alcohol consumption	Yes	Ref			
	No	−25.35	.72		
Feeding frequency	≤3 times/ day	Ref			
	>3 times/ day	−17.08	.78		
GWG	Low	Ref			
	Normal	71.84	.16	61.64 (−44.86, 168.15)	.26
	High	113.34	.43	125.14 (−150.58, 400.87)	.37
Anemia status	Non‐anemic	Ref			
	Anemic	241.19	.06	190 (−72.53, 453.23)	.16
WDDs	Low	Ref			
	High	59.15	.43		
Mid‐upper‐arm circumference (cm)	<21	Ref			
	21–23	191.56	.02	187.10 (32.76, 341.44)	.02*
	> 23	317.5	<.0001	272 (118.39, 425.61)	.001*
Gestational age at delivery (in weeks)		10.09	.02	12.58 (4.1, 21.06)	.004*
Dietary pattern 1		70.5	.004	52.45 (4.46, 100.45)	.03*
Dietary pattern 2		49.75	.04	66.76 (16.56, 116.97)	.01*
Dietary pattern 3		20.59	.4		
Overall *R* ^2^ = 16.89% and Adjusted *R* ^2^ = 14.22%					

Abbreviations: CI, confidence interval; CS, cesarean section; GWG, gestational weight gain; Hgb, hemoglobin; MUAC, mid‐upper‐arm circumference; Ref, reference group; WDDs, Women Diet Diversity Scores; STDs, sexually transmitted diseases. *p < .05 and **p < .001.

## 4 DISCUSSION

This study tried to assess the maternal dietary pattern and its association with birthweight among pregnant mothers in their third trimester of pregnancy attending their routine antenatal care service in Northern Ethiopia. The major staple foods in Adigrat are teff, barley, other cereals like corn and wheat, agricultural products like cereals, grains, roots, vegetables, and fruits, and animal products like meat, milk and milk products, and poultry are consumed by the majority of the population (Mengesha et al., 2017).

Based on the data collected using the FFQ, three distinct dietary patterns were identified. Dietary pattern 1 included egg, milk and milk products, and certain fruits and roots. Dietary pattern 2 included certain vegetables and drinks like coffee, tea, and soda. Dietary pattern 3 had higher loading factors for nuts, grains, and meat. In our study, we have identified three dietary patterns. Although, until recently, there was little research on women's food habits during pregnancy, particularly in Sub‐Saharan Africa, those studies did find several dietary patterns that were almost identical to those seen in our investigations (Tshotetsi et al., 2019). For instance, two dietary patterns that can be classified as health‐conscious and non‐health‐conscious were found in a study of pregnant women in Ghana (Abubakari & Jahn, 2016).

Three dietary patterns—animal‐based, plant‐based, and grain‐based—were discovered in another study conducted in Malawi among pregnant HIV‐positive women (Ramlal et al., 2015). Four dietary patterns—animal‐based, staple‐based, recommended diet, egg and breakfast cereals, legumes, and vegetables—were also discovered in a second study on the HIV‐infected population in South Africa (Annan et al., 2015). A study on Danish women also identified three categories of food habits: western, intermediate, and health‐conscious patterns (Knudsen et al., 2008).

Another study among expectant New Zealanders found three patterns: traditional, fusion, and junk diets (Thompson et al., 2010). But Mexican‐American women (Wolff & Wolff, 1995) and Finnish women (Arkkola et al., 2008) and each identified seven eating patterns. In contrast, these studies employed a lower factor loading of 0.2 as opposed to 0.3, which is used by others, including the current study. This study is consistent with the majority of studies on maternal food habits that showed a connection between the patterns found and birthweight. According to this study, there is a substantial link between the two dietary patterns that include fruits, eggs, milk, vegetables, and other protein‐rich foods and an infant's birthweight increase. This was corroborated by a study on Mexican‐American women, which showed that nutrient‐dense, protein‐rich meals were linked to higher infant birthweight (Wolff & Wolff, 1995).

In addition, there were trends toward associations between a higher VFR (vegetable, fruit, and rice) pattern score and higher birthweight in a study conducted in China (Chia et al., 2019) similar to ours. Overall, it found that healthier diets classified as ones containing eggs, milk, fruits, and vegetables consistently produced superior results in terms of birthweight, as seen in this study (Chia et al., 2019). There are several potential explanations for the link between the increasing consumption of fruits, vegetables, and foods high in protein and birthweight.

Several essential minerals, including potassium, magnesium, dietary fiber, folate, and vitamins A and C, are mostly found in fruits and vegetables (Knudsen et al., 2008). A diet high in fruits and vegetables supports healthy immunological and placental processes, both of which are crucial for the development of the fetus (Kliegman et al., 2016). Alternatively, increased fruit and vegetable intake may simply be a marker for healthier dietary patterns or a healthier lifestyle that could not be fully adjusted in this study. While other studies have shown an association between cereal and red meat‐based diets with lower birthweight outcomes in infants (Knudsen et al., 2008), our study has identified no significant association.

This could be due to a small variation between the mothers in the intake of cereals because almost all mothers in our study consumed cereals daily. Although in the studies cereal was consumed on a daily base by almost all mothers similar to our study, the foods were adjusted using portion size, unlike our study. However, our dietary pattern 1 had a very low (negative) loading factor for grains and sweetened beverages and alcohol, which is associated with higher birth and weight outcomes.

High adherence to dietary patterns 1 and 2 was found to significantly increase the likelihood of having an infant with a higher birthweight. A 1 unit increase in the loadings of dietary pattern 1 was linked to an increase in birthweight of 52.45 g, while a 1 unit increase in the loadings of dietary pattern 2 was linked to an increase in birthweight of 66.76 g. Similar results were found in earlier investigations, which indicated that residence also appeared to be significantly related to birthweight (Mengesha et al., 2017). In a study conducted in Tigray, mothers living in a rural area were at increased risk of delivering low‐birthweight infants (Mengesha et al., 2017) similar to this study. This similarity is because the study area is similar to our study. Furthermore, due to limited awareness and inadequate use of health services, rural mothers are more likely to deliver babies with low birthweight (World Health Organization & United Nations Children's Fund, 2004).

These inadequacies could put these women at risk of receiving subpar prenatal care and related services. Additionally, these ladies may not be getting enough nourishment because they live in a remote area, as most studies have found that rural residents are not getting enough nutrition (Islam, 2015). Additionally, there was a strong correlation between birthweight and gestational weight increase. This was supported by a study done in Mekelle private clinics, which has shown that a 1 unit increase in weight gain during pregnancy was associated with a 0.48 increase in birthweight (Tela et al., 2019).

This could be due to the major contribution weight gain during pregnancy makes to intrauterine fetal growth, which is an important measure of prenatal nutrition (Kliegman et al., 2016). Similar to this, a within‐family analysis conducted in the United States found that inadequate GWG increases the likelihood of low birthweight, whereas excessive weight gain considerably raises the probability of having high‐birthweight babies (Goldstein et al., 2017). This similarity may result from the fact that no single item can satisfy all of a person's nutritional needs, therefore the more food categories one consumes daily, the more likely they are to meet those needs (Dewey, 2016).

Therefore, a sufficiently diverse diet may reflect nutrient adequacy (Abubakari & Jahn, 2016) and increased birthweight. In addition, gestational age at delivery was also significantly associated with birthweight. Similar to our study, prematurity was significantly associated with birthweight in previous findings from Iran (Chaman et al., 2013) and Kenya (Muchemi et al., 2015). According to a study conducted in the Tigray region of Ethiopia, preterm delivery was nearly three times as significantly associated with macrosomia as post‐term birth was with low birthweight (Mengesha et al., 2017). Additionally, in a study done in Northern Ghana, longer gestation was linked to heavier babies; each additional week of gestation was linked to a 0.141 standard‐unit increase in birthweight (Abubakari & Jahn, 2016).

The strength of this study is that it assessed the effect of food interactions on birthweight which a single nutrient analysis may not be able to identify. In addition, we paid attention to environmental, behavioral, and lifestyle factors, as well as other nutritional and pregnancy‐related factors, in addition to sociodemographic characteristics. It is also important to note the study's eligibility requirements, which call for healthy women who do not have any of the conditions listed in the protocol (such as diabetes mellitus and hypertension) and do not require particular dietary regimens. Dietary intake was evaluated before the outcomes were known, which is another significant strength.

The limitation of this study is predominantly that the FFQ used to collect data was not an FFQ specifically prepared for this study population. Each population has its dietary habits. Age, socioeconomic standing, racial or ethnic origin, cultural practices, and access to food can all affect them (Northstone et al., 2008). Therefore, for this limitation, a modified Helen Keller FFQ for vitamin A assessment was used. Additionally, there are flaws in every nutritional assessment technique. FFQs are inaccurate and prone to underreporting.

In addition, because we only looked at the previous week's food intake, we cannot guarantee that the data are indicative of dietary patterns throughout the entire pregnancy. But prior research has indicated that pregnant women's overall food habits stay the same (Northstone et al., 2008). Thirdly, as information on portion size was not gathered in the current study and dietary consumption was mainly based on the frequency of food intake, we were unable to account for total calorie intake. Last but not least, despite making multiple statistical adjustments, residual confounding associated with fetal growth and diet during pregnancy cannot be completely ruled out.

## 5 CONCLUSIONS

In conclusion, three dietary patterns were identified; of those patterns, dietary pattern 1 and dietary pattern 2 were significantly associated with birthweight. Women who consumed more eggs, milk and milk products, pulses, vegetables and roots, fruits, beverages, and sweets had an increased birthweight outcome. Therefore, intrauterine growth conditions in the third trimester might be improved by adopting certain dietary patterns during pregnancy, especially dietary patterns dense with egg, milk, and milk products, fruits, vegetables and roots, and pulses.

## AUTHOR CONTRIBUTIONS

**Selamawit Gebreyohannes Weldegebriel:** Conceptualization (equal); funding acquisition (equal); methodology (equal); project administration (equal); supervision (equal); writing – original draft (equal); writing – review and editing (equal). **selamawit Asfaw Beyene:** Conceptualization (equal); supervision (equal); writing – review and editing (equal). **Freweini Gebrearegay Tela:** Conceptualization (equal); supervision (equal); writing – review and editing (equal). **Zenawi Hagos Gufue:** Software (equal); supervision (equal); writing – original draft (equal); writing – review and editing (equal). **Helen Teweldebirhan Hailu:** Software (equal); supervision (equal); writing – original draft (equal); writing – review and editing (equal).

## Data Availability

The datasets generated and analysed during the current study are available from the corresponding author on a reasonable request.
